# Topoisomerase 1 Regulates Gene Expression in Neurons through Cleavage Complex-Dependent and -Independent Mechanisms

**DOI:** 10.1371/journal.pone.0156439

**Published:** 2016-05-27

**Authors:** Angela M. Mabb, Jeremy M. Simon, Ian F. King, Hyeong-Min Lee, Lin-Kun An, Benjamin D. Philpot, Mark J. Zylka

**Affiliations:** 1 Department of Cell Biology and Physiology, UNC Neuroscience Center, Carolina Institute for Developmental Disabilities, The University of North Carolina, Chapel Hill, North Carolina, United States of America; 2 Neuroscience Institute, Georgia State University, Atlanta, Georgia, United States of America; 3 School of Pharmaceutical Sciences, Sun Yat-sen University, Guangzhou, China; University of Louisville, UNITED STATES

## Abstract

Topoisomerase 1 (TOP1) inhibitors, including camptothecin and topotecan, covalently trap TOP1 on DNA, creating cleavage complexes (cc’s) that must be resolved before gene transcription and DNA replication can proceed. We previously found that topotecan reduces the expression of long (>100 kb) genes and unsilences the paternal allele of *Ube3a* in neurons. Here, we sought to evaluate overlap between TOP1cc-dependent and -independent gene regulation in neurons. To do this, we utilized *Top1* conditional knockout mice, *Top1* knockdown, the CRISPR-Cas9 system to delete *Top1*, TOP1 catalytic inhibitors that do not generate TOP1cc’s, and a TOP1 mutation (T718A) that stabilizes TOP1cc’s. We found that topotecan treatment significantly alters the expression of many more genes, including long neuronal genes, immediate early genes, and paternal *Ube3a*, when compared to *Top1* deletion. Our data show that topotecan has a stronger effect on neuronal transcription than *Top1* deletion, and identifies TOP1cc-dependent and -independent contributions to gene expression.

## Introduction

Topoisomerases are enzymes that resolve DNA supercoils by creating transient single (Type I topoisomerases) or double (Type II topoisomerases) strand breaks [1,2]. These enzymes facilitate DNA replication, chromosomal segregation, DNA repair, and gene transcription [3]. In postmitotic cells, topoisomerases predominantly regulate gene transcription and DNA repair [4]. Topoisomerase I (TOP1) relieves DNA supercoiling ahead of RNA polymerase to facilitate transcription elongation [5–7]. Although the roles of topoisomerases in dividing cells have been studied extensively, much less is known about their functions in neurons.

Long noncoding RNAs (lncRNA) can act as transcriptional activators or repressors in postmitotic neurons and other cell types [8,9]. *Ube3a* antisense (*Ube3a-ATS*) is an extremely long lncRNA (> 1 Mb) and is expressed exclusively from the paternal allele in most neurons during development and throughout adulthood. Paternal expression of *Ube3a-ATS* silences the paternal copy of *Ube3a* via a transcriptional collision mechanism [10–12].

We previously found that TOP1 and TOP2 inhibitors unsilence the paternal allele of *Ube3a* in postmitotic neurons by reducing expression of *Ube3a-ATS* [13]. Mutations that reduce or increase UBE3A function are linked to Angelman syndrome (AS) and autism, respectively [14–21]. In addition to downregulating *Ube3a-ATS*, TOP1 and TOP2 inhibitors also downregulate the expression of other long (generally >100 kb) genes in neurons, many of which are associated with neurotransmission and synaptic function [22]. Consistent with reduced expression of long synaptic genes, inhibition of TOP1 with topotecan disrupts excitatory and inhibitory synaptic transmission in cortical neuron cultures, an effect that is reversible following inhibitor washout [23]. TOP1 inhibitors also reduce expression of long genes in non-neuronal cell types [24,25].

Topotecan binds at the interface between TOP1 and DNA, creating a TOP1-DNA enzyme intermediate known as a TOP1 cleavage complex (TOP1cc) [26]. Given this unique mechanism of inhibition, we sought to determine the extent to which TOP1 and TOP1cc formation contribute to neuronal gene expression and *Ube3a* regulation. To answer these questions, we generated a *Top1* conditional knockout mouse to genetically delete *Top1* from cultured neurons. We also utilized the CRISPR-Cas9 system to delete *Top1*, used short hairpin (sh)RNAs to knock-down *Top1*, compared TOP1 catalytic inhibitors that do not generate TOP1cc’s to topotecan, and utilized a TOP1 (T718A) mutation that stabilizes TOP1cc’s. Surprisingly, we found that topotecan affected the expression of many more genes when compared to deletion of *Top1*—the molecular target of topotecan. Taken together, our findings reveal TOP1cc-dependent and -independent control of gene expression and *Ube3a* regulation in neurons. Our findings also have implications for cancer therapies that target TOP1 via these distinct mechanisms.

## Materials and Methods

Knockout first ES cells targeting the *Top1* gene were acquired from the KOMP Repository Knockout Mouse Project (Project ID: CSD36970, *Top1*^*tm1a(KOMP)Wtsi*^). ES cells were microinjected into albino C57BL/6 blastocysts by the UNC Animal Models Core Facility. Two chimeric lines were bred for germline transmission. Successful germline transmitted mice were then crossed to a *FLP1* recombinase deleter mouse B6.Cg-Tg(ACTFLPe)9205Dym/J (Jackson Laboratory) to excise the *lacZ/neomycin* cassette (removal confirmed by PCR), then backcrossed further to C57Bl/6 mice to remove the Flp transgene. To distinguish genotypes for *Top1* cKO mice, the following primers flanking the LoxP site and within the *Top1* gene were used: geno 2, 5’-GAGTTTCAGGACAGCCAGGA-3’ and geno 3, 5’-GGACCGGGAAAAGTCTAAGC-3’.

### Neuronal Cultures

Embryonic day E13.5–15.5 mouse cortical neuron cultures were prepared by cervical dislocation of adult C57BL6/J wild-type females as described [13]. Animals were kept on a 12-hour light-dark cycle and given *ad libitum* access to food and water. All experimental animal procedures were carried out according to the NIH *Guide for the Care and Use of Laboratory Animals* and were approved by the Institutional Animal Care and Use Committee at the University of North Carolina at Chapel Hill. For immunostaining, dissociated neurons were plated in 24-well dishes containing poly-D-lysine (0.1 mg/ml) coated 12 mm coverslips at a density of 2.5 x 10^5^ cells/well. For biochemistry, dissociated neurons were seeded on poly-D-lysine coated 12-well dishes at a density of 5 x 10^5^ cells/well.

### Western Blotting

Lentiviruses harboring pLenti-CaMKIIα-tdTomato and pLenti-CamKIIα-tdTomato-P2A-CRE based vectors were prepared by the UNC Lentiviral Core. Lentiviral *Top1* shRNA was generated as previously described [22]. Briefly, cortical neurons were transduced at DIV 3 with lentivirus at a multiplicity of infection of at least two to maximize the number of transduced cells (around 85–90% transduction efficiency). Media containing lentivirus was removed 24 hours later and replaced with conditioned media. The CaMKIIα promoter limited tdTomato expression to neurons and was detectable without antibody amplification 3–4 days post transduction. Neurons were then treated at DIV 15 with vehicle (0.003% DMSO, Neurobasal medium) or 300 nM topotecan (Molcan Corporation; in 0.003% DMSO, Neurobasal medium) and harvested 3 days later.

For western blot experiments, cells were harvested and lysed in RIPA buffer (50 mM Tris-HCl, 150 mM NaCl, 0.5% sodium deoxycholate, 1% Triton X-100, and 0.1% SDS, pH 7.4) with 1 mM DTT, 1 μg/mL aprotinin, 2 μg/mL leupeptin, and 0.1 mM PMSF. Total protein (25–40 μg) was run on a 4–15% gradient SDS-PAGE gel (Bio-RAD). Proteins were then transferred to nitrocellulose membrane, blocked overnight in Odyssey Blocking Buffer (LI-COR), and immunoblotted overnight using the following antibodies: rabbit anti-UBE3A (1:1,000; Bethyl Laboratories, A300-352A), mouse anti-UBE3A (1:1,000; BD Biosciences), mouse anti-NLGN1 (1:500; Synaptic Systems, 129 111), mouse anti-NRXN1 (1:500; BD Biosciences, 611882), mouse anti-CNTNAP2 (1:1,000; NeuroMab, 75–075), mouse anti-β-actin (1:5,000; Millipore, MAB1501R), rabbit anti-TOP1 (1:10,000; GeneTex, GTX63013), or mouse anti-TOP1 (1:250; Santa Cruz, sc-271285). The GeneTex rabbit monoclonal antibody was raised against the N-terminus of human TOP1. The Santa Cruz mouse monoclonal antibody was raised against the C-terminus (amino acids 685–765) of human TOP1. Both antibodies are predicted to react with mouse TOP1. Membranes were washed three times with water at room temperature and the appropriate IRDye secondary antibodies (Li-COR) were added at a dilution of 1:15,000–1:20,000 for 1 hour at room temperature. Blots were then washed two times in Tris-buffered saline containing 0.1% Tween-20 and two times with water. Membranes were dried in the dark and imaged using the ODYSSEY CLx Infrared Imaging System (LI-COR). Equivalent amounts of protein per sample were loaded and loading controls were used to ensure equivalent loading between samples. Experiments were performed on a minimum of three independent culture sets.

### Immunocytochemistry

Neurons were fixed for 20 min in cold phosphate buffered saline (PBS) containing 4% paraformaldehyde and 4% sucrose. Cells were permeabilized with 0.1% Triton X-100 in PBS for 15 min at room temperature and blocked in 10% normal goat serum (NGS) for 1 h at 37°C [27]. Neurons were then incubated with 1:1,000 mouse anti-UBE3A (Sigma, clone #330) and 1:1,000 rabbit anti-TOP1 (Genetex) in 3% NGS overnight at 4°C. Cells were washed three times with 3% NGS in PBS and incubated with a 1:1,000 dilution of Alexa dye-conjugated secondary antibodies (Invitrogen) and 1:10,000 dilution of DAPI (ThermoFisher Scientific) in 3% NGS in PBS in the dark for 1 hour at room temperature. Cells were then washed three times with PBS and mounted on slides using FluoroGel mounting media (Electron Microscopy Sciences). To detect TOP1 DNA covalent complexes, we utilized an antibody that specifically recognizes TOP1-DNA covalent complexes [27]. Briefly, neurons were fixed as stated above, permeabilized in 0.25% Triton X-100 in PBS for 15 min at room temperature, washed 3 times in 0.1% Triton X-100, and then blocked in 10% NGS in PBS for 1 h at 37°C. Neurons were incubated with 1:1,500 dilution of mouse anti-TOP1-DNA covalent complex antibody (Millipore, clone 1.1A) in 3% NGS in PBS overnight at 4°C.

Images were acquired using a Zeiss LSM 710 upright microscope with a 20X/0.8 NA objective. Images were acquired with identical settings (gain, contrast, pinhole) for UBE3A, TOP1, and TOP1 DNA covalent complexes. Treatment and transfected conditions were interleaved during each imaging session. The intensity of UBE3A, TOP1, and TOP1 DNA covalent complexes was quantified from maximum intensity projections in FIJI following thresholding at least 2 standard deviations above background. For the CRISPR-Cas9 *Ube3a* unsilencing experiments, regions of interest (ROIs) were identified by outlining the soma of tdTomato positive neurons manually. ROIs were then transposed on both the UBE3A and TOP1 channel to measure the integrated density. Note that for the *Top1* CrispR-Cas9 experiments, we excluded cells where TOP1 was not deleted, and average TOP1 integrated density was at or above the average TOP1 intensity in untransfected neurons. For experiments using *AS*::*Top1*^*fl/fl*^ neuron cultures, ROIs were selected automatically in FIJI. Briefly, DAPI images were thresholded and nuclei were separated using the Watershed Tool. ROIs were then outlined using the Analyze Particles tool with a setting size of 8 μm to infinity. The integrated density of each ROI was then transposed to a thresholded (two standard deviations above background) UBE3A channel where the integrated density was measured. ROIs were then transposed onto the GFP channel to identify transfected and untransfected neurons.

### Cloning

To generate the pLenti-CamKIIα-tdTomato and pLenti-CamKIIα-tdTomato-P2A-CRE constructs, tdTomato and tdTomato-P2A-CRE fragments were PCR cloned into the pLenti-CamKIIα-ChR2-mCherry vector (http://www.everyvector.com/sequences/show/20437). Briefly, ChR2-mCherry was excised and replaced with tdTomato or td-Tomato-P2A-CRE using *AgeI* and *BsrGI* sites for tdTomato and BamHI and EcoRI sites for tdTomato-P2A-CRE. Human GFP-TOP1 was PCR cloned into the the pLenti-CamKIIα-ChR2-mCherry vector (modified from Karl Deisseroth’s laboratory) using *AgeI* and *EcoRI* restriction sites. The PCR template for human TOP1 was a kind gift from Stefan Weger from the Intitut fur Virologie in Berlin, Germany. The GFP-TOP1 cleavable complex mimetic (T718A) was created using site directed mutagenesis using the following primer sets: 5’-AAACAGATTGCCCTGGGAGCCTCCAAACTCAATTATC-3’ and 5’-GATAATTGAGTTTGGAGGCTCCCAGGGCAATCTGTTT-3’. CRISPR-Cas9 targeting of *Top1* was accomplished by annealing *Top1* sgRNAs into the lentiCRISPR v1 vector backbone (Addgene) using the suggested cloning strategy (http://www.genome-engineering.org/crispr/?page_id=23). A total of four *Top1* sgRNA targets were designed using the E-CRISP design tool (http://www.e-crisp.org/E-CRISP/). The following primer sets were used to clone into the lentiCRISPR v1 backbone: **Clone #1:** 5’-CACCGCCGGGGCTTTTCCGAGGCCG-3’ and 5’-AAACCGGCCTCGGAAAAGCCCCGGC-3’ **Clone #2:** 5’-CACCGATCGGAAATCCGCTTCGATC-3’ and 5’-AAACGATCGAAGCGGATTTCCGATC-3’ **Clone #3:** 5’-CACCGTCGGAAATCCGCTTCGATCT-3’ and 5’-AAACAGATCGAAGCGGATTTCCGAC-3’ **Clone #4:** 5’-CACCGAGATCGAGAACACCGGCATA-3’ and 5’-AAACTATGCCGGTGTTCTCGATCTC-3’. Each individual clone was tested for *Top1* loss by immunostaining for TOP1 protein in neurons. Clone #1 and #4 were deemed the most efficient; clone #4 was used for subsequent experiments.

### RNA-seq

*Top1*^*fl/fl*^ neurons were infected with either tdTomato or tdTomato-P2A-CRE lentivirus and treated as stated above. RNA was isolated with the RNeasy plus mini kit (Cat. #74134, Qiagen). RNA yield and quality was determined with a Nanodrop 1000 Spectrophotometer (Thermo Scientific). Samples were further assessed for quality using either an Agilent Bioanalyzer 2100 or TapeStation 2200 to obtain a RNA integrity number (RIN). RIN values exceeding 7 were used for sequencing. RNA samples were used to generate and barcode cDNA libraries using the TruSeq RNA Library Preparation Kit at the UNC High Throughput Sequencing Facility. Pools of 24 multiplexed samples were sequenced per lane in a HiSeq 2500 sequencer using 50 bp paired-end reads.

### RNA-Seq Data Processing

RNA-seq reads were filtered using TagDust and aligned to the reference mouse genome (mm9) with TopHat using default parameters. Reads aligning to rRNA genes were removed. Transcript abundance was estimated by computing RPKM using RefSeq gene models aggregated by gene symbol. For differential expression analyses, raw counts over RefSeq exons were used and compared across samples using EdgeR. RNA-seq data were deposited in the GEO database (accession no. GSE79951).

### Synthesis of TOP1 Catalytic Inhibitors

TOP1 catalytic inhibitors were synthesized and characterized as described [28–30].

## Results

### TOP1-Dependent Control of Neuronal Genes

**Fig 1:**
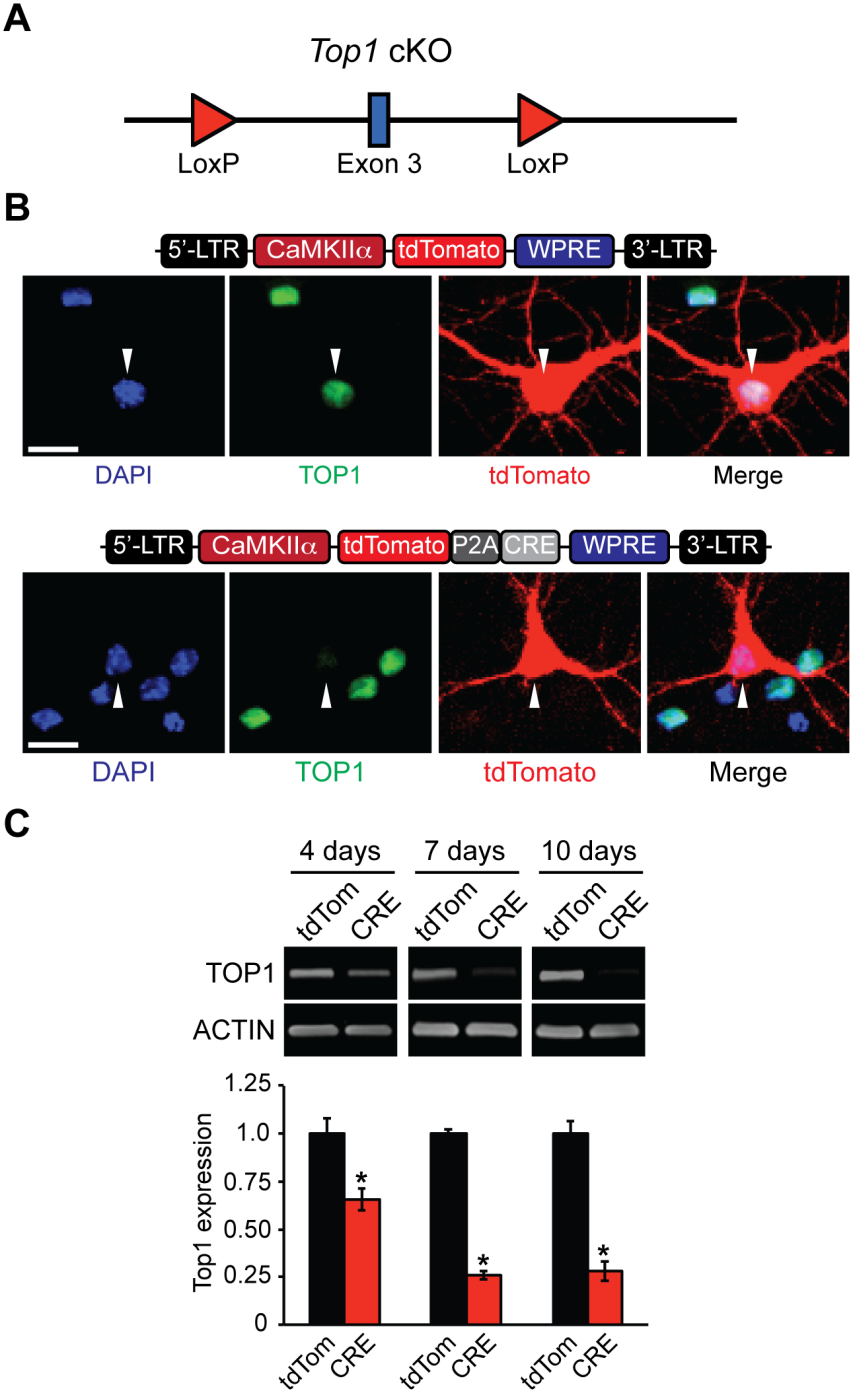
Generation and validation of *Top1* cKO mouse. (A) Schematic of the *Top1* cKO allele. LoxP sites flank *exon 3*. (B) Schematic of tdTomato (top) and tdTomato-P2A-CRE lentiviral plasmids (bottom). *Top1*^*fl/fl*^ neurons were transfected with tdTomato or tdTomato-P2A-CRE plasmids. Neurons were fixed and immunostained with an anti-TOP1 antibody. Scale bar, 10 μm. (C) Cortical neurons were infected with tdTomato or tdTomato-P2A-CRE lentivirus at DIV 3 and then were harvested at DIV 7, DIV 10, and DIV 13. Representative immunoblots and quantification of TOP1 protein expression normalized to ACTIN (bottom). Mean ± s.e.m., unpaired student’s t-test; * p < 0.05, n = 3 cultures.

The TOP1 inhibitor topotecan suppresses expression of long genes and unsilences the paternal copy of *Ube3a* in neurons [13,22]. To determine if these transcriptional effects could be recapitulated by deletion of *Top1*, we generated a *Top1* conditional knockout mouse (cKO), as homozygous deletion of *Top1* is embryonic lethal with failure occurring between the 4 and 16-cell stages [31]. The *Top1* cKO allele contains two LoxP sites flanking exon 3 (Fig 1A) such that Cre-mediated excision is predicted to facilitate nonsense-mediated decay of *Top1* mRNA and thus disrupt TOP1 protein levels. To confirm that *Top1* can be deleted in these mice, we created tdTomato (control) and CRE-dependent lentiviral constructs driven by the neuron-specific CamKIIα promoter (Fig 1B). Transfection of CRE, but not tdTomato, reduced TOP1 protein levels in *Top1* cKO neurons (Fig 1B). Infection of *Top1*^*fl/fl*^ cultured neurons with tdTomato or CRE lentivirus resulted in a transduction efficiency ranging from 85–90% (data not shown). TOP1 levels were maximally decreased 7 days post infection with CRE compared to tdTomato control neurons (Fig 1C). Additionally, no lower molecular weight TOP1-reactive bands were detected, indicating that truncated products of TOP1 are not generated in neuronal cultures from *Top1* cKO mice (S1A Fig). The residual levels of TOP1 most likely originate from uninfected neurons and/or non-neuronal cells in the cultures.

**Fig 2:**
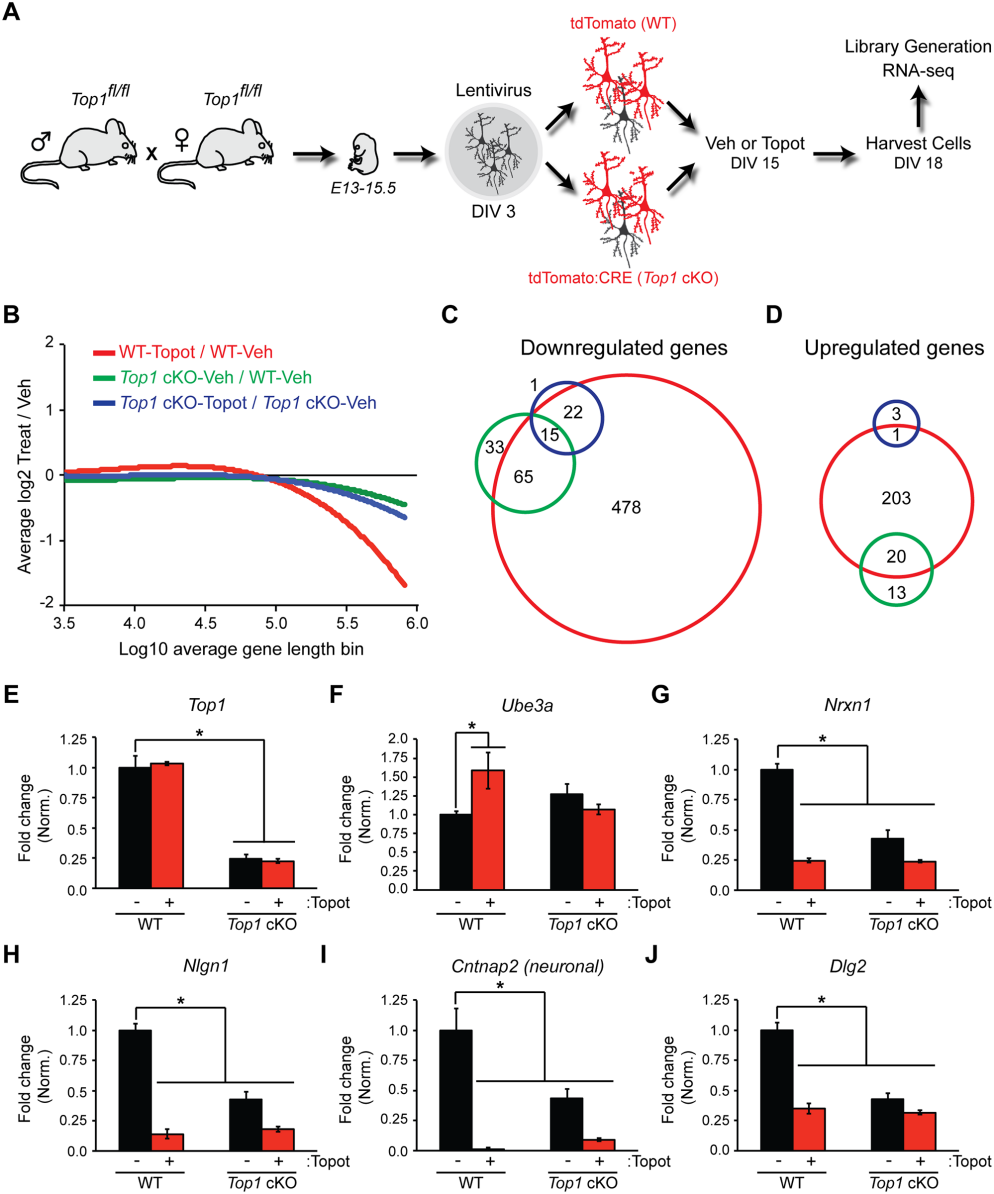
*Top1* deletion is necessary and sufficient for the expression of some long genes. (A) Schematic of the experimental setup used to assess changes in transcript levels following *Top1* deletion. (B) LOESS smoothing curve showing RNA-seq average log2 change in transcript levels in topotecan-treated WT (WT-Topot, red), vehicle-treated *Top1* cKO (*Top1* cKO-Veh, green), and topotecan-treated *Top1* cKO (*Top1* cKO-Topot, blue) cortical neuron cultures relative to vehicle-treated WT (WT-Veh) cells plotted in bins of 200 genes by length. (C) Venn diagram showing the number of significantly downregulated genes in topotecan-treated WT (WT-Topot, red), vehicle-treated *Top1* cKO (*Top1* cKO-Veh, green), and topotecan-treated *Top1* cKO (*Top1* cKO-Topot, blue) cortical neuron cultures relative to vehicle-treated WT (WT-Veh) cells. The FDR was set at a value of < 0.01. (D) Venn diagram showing the number of significantly upregulated genes. (E—J) Representative transcript level changes from RNA-seq analysis. Normalized RPKM values (relative to WT-Veh) in WT-Veh, WT-Topot, *Top1* cKO-Veh, and *Top1* cKO-Topot. Mean ± s.e.m., FDR < 0.1, n = 3 cultures.

To determine how *Top1* deletion affects neuronal gene expression relative to topotecan, we infected *Top1*^*fl/fl*^ cortical neuron cultures with tdTomato control (WT) or CRE (*Top1* cKO) and measured changes in transcript levels via RNA-seq in cells treated with vehicle (Veh) or topotecan (Topot) (Fig 2A). As expected, topotecan-treated WT cells (WT-Topot) exhibited global reductions in the expression of long genes compared to vehicle-treated WT cells (WT-Veh). Vehicle-treated *Top1* cKO (*Top1* cKO-Veh) cells also exhibited global reductions in the expression of long genes compared to WT-Veh cells (Fig 2B), although the effect size was attenuated when compared to topotecan-treated cells (Fig 2B). Topotecan treatment did not further alter the expression of long genes in *Top1* cKO cells (*Top1* cKO-Topot; Fig 2B), suggesting the transcriptional effects of topotecan depend on *Top1* and are thus molecularly on-target.

Relative to WT-Veh, we found multiple downregulated (Fig 2C) and upregulated (Fig 2D) genes (FDR of < 0.01) in WT-Topot, *Top1* cKO-Veh, and *Top1* cKO-Topot cortical neuron cultures. A total of 580 genes were significantly decreased in WT-Topot cells, whereas 113 genes were significantly decreased in *Top1* cKO-Veh cells. Eighty of these 113 genes were downregulated in both WT-Topot and *Top1* cKO-Veh cells (S1 Table). Based on Gene Ontology, downregulated genes in WT-Topot and *Top1* cKO-Veh cells were functionally annotated to common biological processes such as synaptic transmission and cell adhesion (S2 Table). There was no statistically identifiable functional annotation for *Top1* cKO-Topot cells in the downregulated gene set (S2 Table). We also looked at the expression of individual genes that were reduced in WT-Topot cells but not in *Top*1 cKO-Veh cells. Strikingly, a large proportion of immediate early genes (IEGs) were decreased in WT-Topot but not in *Top1* cKO-Veh cells (S2 Fig). Moreover, relative to *Top1* cKO-Veh, we did not detect a decrease in IEG expression in *Top1* cKO-Topot cells, suggesting the change in IEG expression is *Top1*-dependent. These findings indicate that topotecan reduces expression of IEGs in a TOP1-dependent manner, and that deletion of TOP1 alone does not reduce expression of IEGs. Collectively, these data indicate that the transcriptional effects of topotecan are significantly greater than the effects of TOP1 deletion, consistent with the fact that topotecan generates TOP1cc’s and does not simply inhibit TOP1.

We found that 224 genes were upregulated in WT-Topot cells, whereas only 33 genes were upregulated in *Top1* cKO-Veh cells. Additionally, *Top1* cKO-Topot cells had a significant increase in 4 genes, of which 1 overlapped with WT-Topot cells (Fig 2D and S1 Table). Based on Gene Ontology, upregulated genes in WT-Topot cells were functionally annotated to axon guidance and cell motion processes where *Top1* cKO-Veh upregulated genes were functionally annotated to eye lens development (S3 Table).

As expected, *Top1* transcript levels were reduced in *Top1* cKO cells (Fig 2E). Moreover, we observed elevated expression of *Ube3a* in WT-Topot but not in *Top1* cKO-Veh cells (Fig 2F). Several long synaptic adhesion genes were also downregulated in WT-Topot and *Top1* cKO-Veh cells (Fig 2G–2J), consistent with our previous findings [22]. Taken together, these data indicate that topotecan-treatment or TOP1 deletion reduces expression of a subset of long genes, which include extremely long synaptic adhesion genes.

### *Top1* Deletion Reduces Synaptic Adhesion Protein Levels

**Fig 3:**
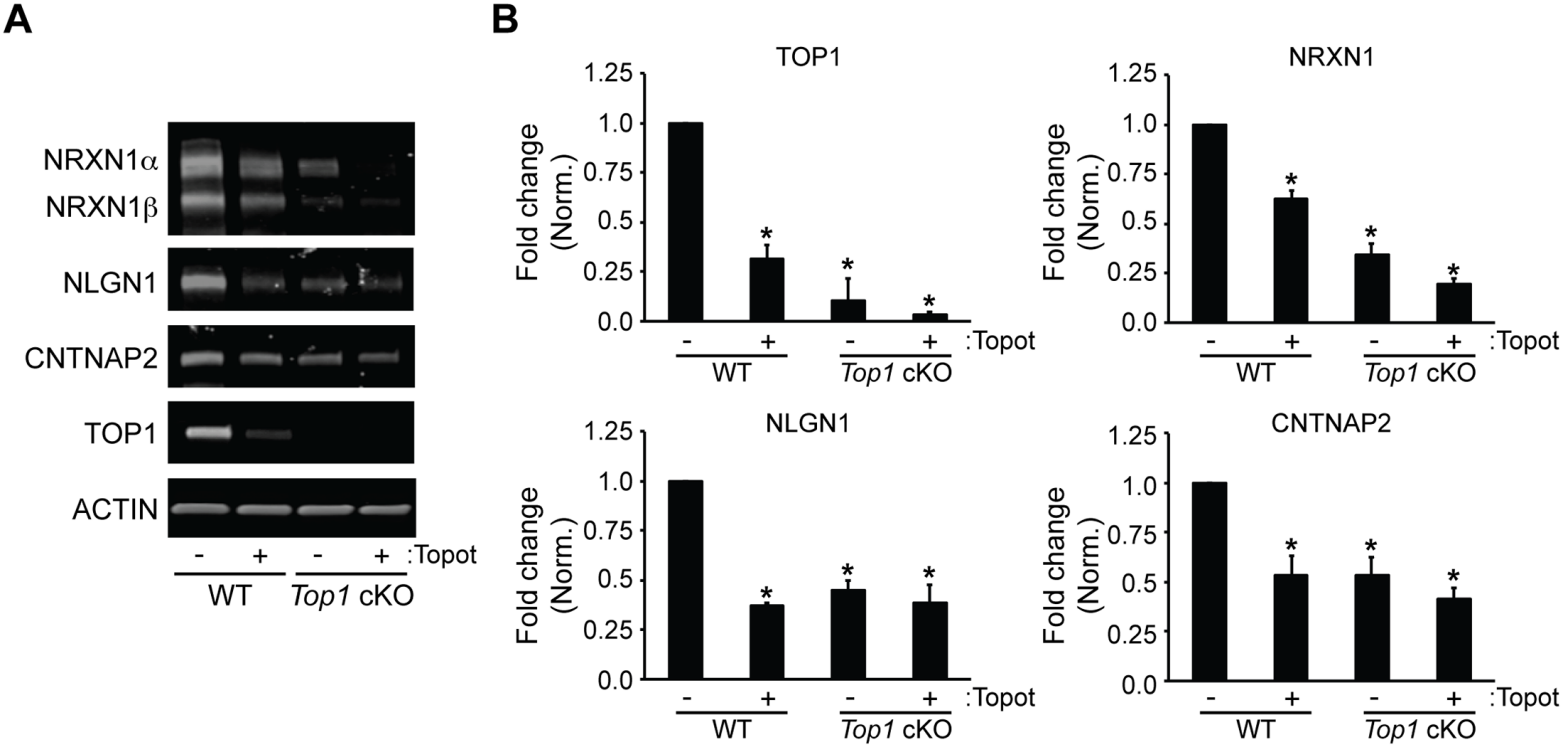
TOP1 deletion reduces synaptic adhesion protein levels. (A) *Top1*^*fl/fl*^ neuron cultures were infected with tdTomato (WT) or tdTomato-P2A-CRE (*Top1* cKO) lentivirus at DIV 3. Cells were then treated at DIV 15 with vehicle (DMSO) or 300 nM topotecan for 72 hours. Shown are representative immunoblots with antibodies to NRXN1, NLGN1, CNTNAP2, TOP1, and ACTIN. (B) Quantification of fold change in TOP1, NRXN1, NLGN1, and CNTNAP2 protein expression normalized to ACTIN. Mean ± s.e.m., unpaired student’s t-test; * p < 0.05, n = 3 cultures.

Previously, we found that topotecan downregulates synaptic proteins and dampens synaptic transmission [23]. Here we found that conditional deletion of *Top1* in cortical neuron cultures reduced the expression of synaptic adhesion proteins to a similar extent as in topotecan-treated WT cells (Fig 3A and 3B). Addition of topotecan to CRE-infected cells did not further decrease protein expression, indicating that *Top1* deletion occluded additional effects of topotecan on these synaptic adhesion proteins (Fig 3A and 3B). Using an independent genetic approach, we employed a *Top1*-specific lentiviral shRNA to reduce TOP1 (S3A and S3B Fig), which also reduced the expression of two long synaptic adhesion proteins, NEUREXIN-1 and NEUROLIGIN-1 (S3A and S3B Fig).

### Topotecan, but Not *Top1* Deletion, Unsilences *Ube3a*

**Fig 4:**
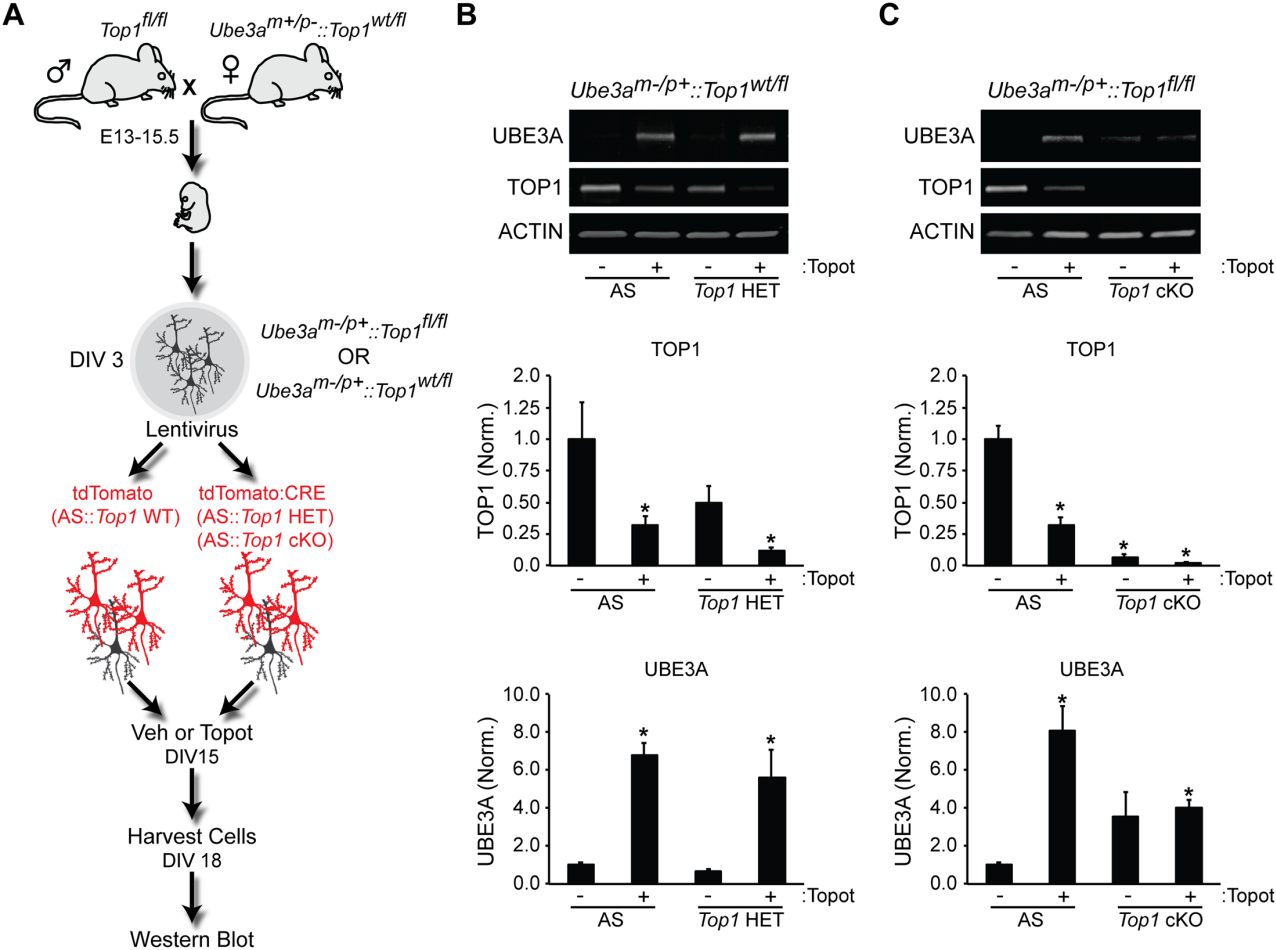
*Top1* deletion does not efficiently unsilence the paternal *Ube3a* allele. (A) Schematic of the experimental setup used to assess *Ube3a* unsilencing. (B,C) Representative immunoblots and quantification of indicated protein levels normalized to ACTIN in neurons from *Ube3a*^*m-/p+*^::*Top1*^*wt/fl*^ mice (B) or *Ube3a*^*m-/p+*^::*Top1*^*fl/fl*^ mice (C). Mean ± s.e.m., unpaired student’s t-test; * p < 0.05, n = 3–4 cultures.

We next sought to determine if genetic reduction or deletion of *Top1* could unsilence *Ube3a* in neurons. First, we cultured cortical neurons lacking the maternal copy of *Ube3a*^*m-/p+*^ (AS) and transduced them with lentiviral *Top1* shRNA (S3C and S3D Fig). This manipulation reduced TOP1 protein levels but was not sufficient to unsilence *Ube3a*, as demonstrated by the lack of detectable paternal UBE3A protein (S3C and S3D Fig). TOP1 protein levels were reduced ~50% in these knockdown experiments, raising the possibility that residual levels of TOP1 might maintain *Ube3a-ATS* transcription and hence maintain repression of paternal *Ube3a*. To examine this possibility, we crossed *Top1* cKO mice with AS mice, prepared cortical neuron cultures, and transduced cells with either tdTomato control (WT) or CRE lentivirus to delete *Top1* (*Top1* cKO) (Fig 4A). Neurons were then treated with vehicle or topotecan to test for the ability to unsilence *Ube3a*. As expected, treatment of *AS*::*Top1*^*wt/fl*^ neurons with topotecan led to unsilencing of paternal *Ube3a* in WT and *Top1* heterozygous mutant neurons (Fig 4B). However, complete deletion of *Top1* did not significantly increase UBE3A levels in *AS*::*Top1*^*fl/fl*^ neurons (Fig 4C). Moreover, compared to WT neurons, *Top1* cKO neurons exhibited blunted *Ube3a* unsilencing after treatment with topotecan (Fig 4C).

**Fig 5:**
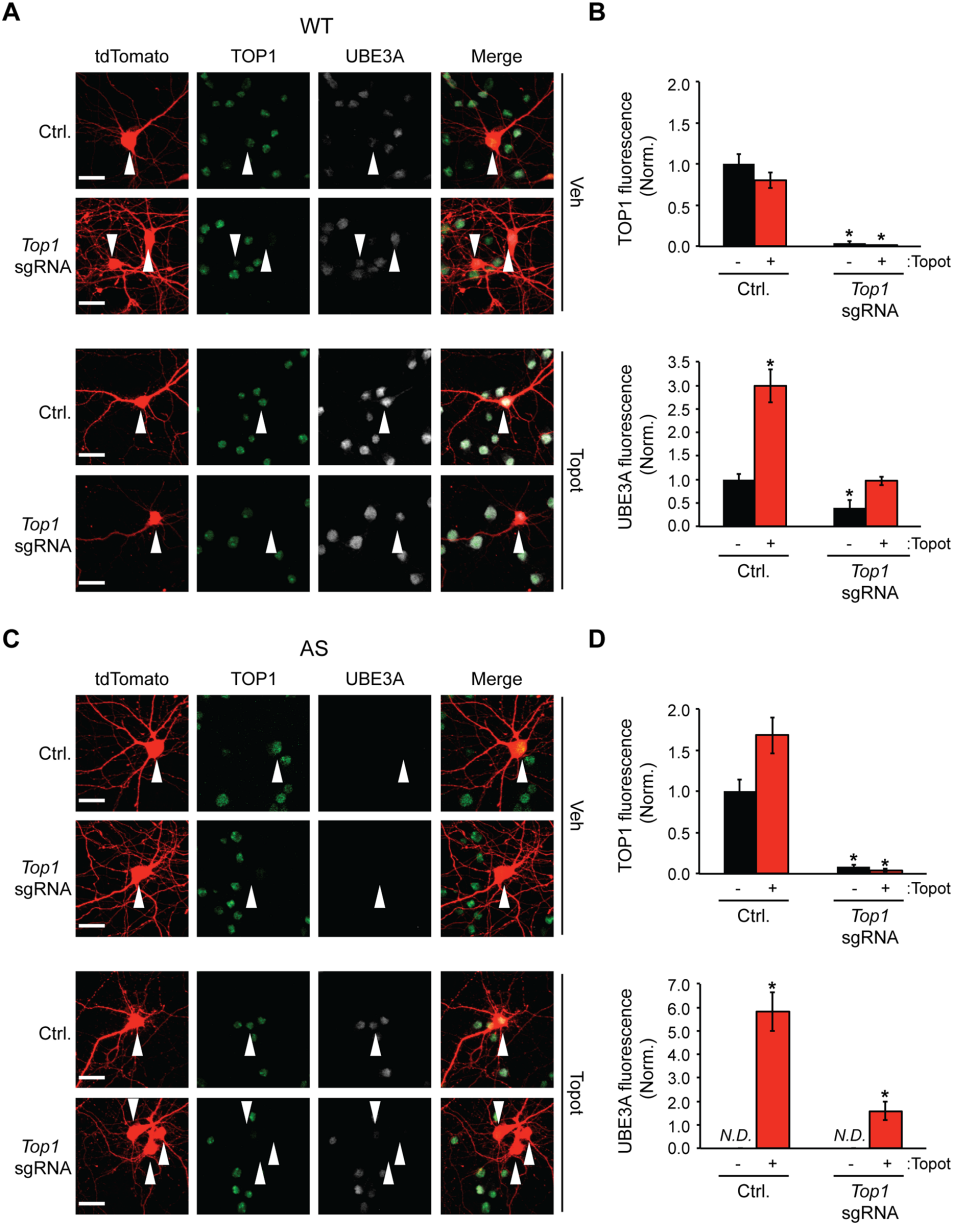
TOP1 is necessary but not sufficient to unsilence *Ube3a*. (A) WT cortical neuron cultures were transfected with tdTomato and Cas9 alone (Ctrl.) or Cas9 and a sgRNA directed to *Top1* at DIV 3. Neurons were then treated with vehicle (DMSO) or 300 nM topotecan for 72 hours. Scale bar, 20 μm. (B) Quantification of TOP1 (top) or UBE3A (bottom) fluorescence. Values were normalized to the fluorescence intensity of control neurons. Mean ± s.e.m., unpaired student’s t-test; * p < 0.05, n = 3 cultures. (C) *Ube3a*^*m-/p+*^ (AS) cortical neuron cultures were transfected with tdTomato and Cas9 alone (Ctrl.) or Cas9 and a sgRNA directed to *Top1* at DIV 3. Neurons were then treated with vehicle (DMSO) or 300 nM topotecan for 72 hours. Scale bar, 20 μm. (D) Quantification of TOP1 (top) or UBE3A (bottom) fluorescence. Values represent raw integrated density values divided by a value of 1000. Mean ± s.e.m., unpaired student’s t-test relative to vehicle-treated Ctrl.; * p < 0.05, n = 3 cultures. *N*.*D*. = Not detected.

To replicate our findings using a different genetic approach, we utilized the CRISPR-Cas9 system to delete *Top1* in wildtype (WT) and AS cortical neuron cultures. WT cells were transfected with Cas9 alone (control) or Cas9 with an sgRNA directed to *Top1*. We observed a near-complete loss of TOP1 using a sgRNA targeted to *Top1* relative to controls (Fig 5A and 5B, S4A Fig). Consistent with our *Top1* cKO studies above, *Top1* CRISPR-mediated deletion did not increase UBE3A expression (Fig 5A and 5B). In contrast, UBE3A levels were increased in topotecan-treated neurons (Fig 5A and 5B), but not in topotecan-treated *Top1* deficient neurons. To examine changes in paternal UBE3A expression, we transfected Cas9 and the sgRNA targeting *Top1* into AS neurons. Consistent with our *Top1* cKO studies above, CRISPR-mediated deletion of *Top1* was not sufficient to increase UBE3A in AS cortical neurons (Fig 5C and 5D). Moreover, the increase in paternal UBE3A expression in topotecan-treated AS neurons was attenuated in topotecan-treated *Top1* deficient neurons (Fig 5C and 5D).

### Formation of TOP1 Cleavage Complexes Unsilences *Ube3a*

Since deletion of *Top1* did not unsilence *Ube3a*, whereas topotecan (which forms TOP1cc’s) did unsilence *Ube3a*, we hypothesized that TOP1cc’s may be required to unsilence *Ube3a* in neurons. To test this hypothesis, we compared topotecan to a series of TOP1 catalytic inhibitors that inhibit TOP1 without forming TOP1cc’s (S5A Fig) [28,29]. As previously found [13], topotecan unsilenced the paternal *Ube3a-YFP allele* in *Ube3a*^*m+/pYFP*^ cortical cultures (S5B and S5C Fig). However, paternal *Ube3a-YFP* was not unsilenced after treating with four different TOP1 catalytic inhibitors, including CYB-L10 [29], over a range of doses (S5B and S5C Fig). CYB-L10 did significantly reduce expression of synaptic adhesion molecules in wildtype cells (S5D and S5E Fig), suggesting the drug can enter cells and reduce expression of genes that are affected by *Top1* deletion.

**Fig 6:**
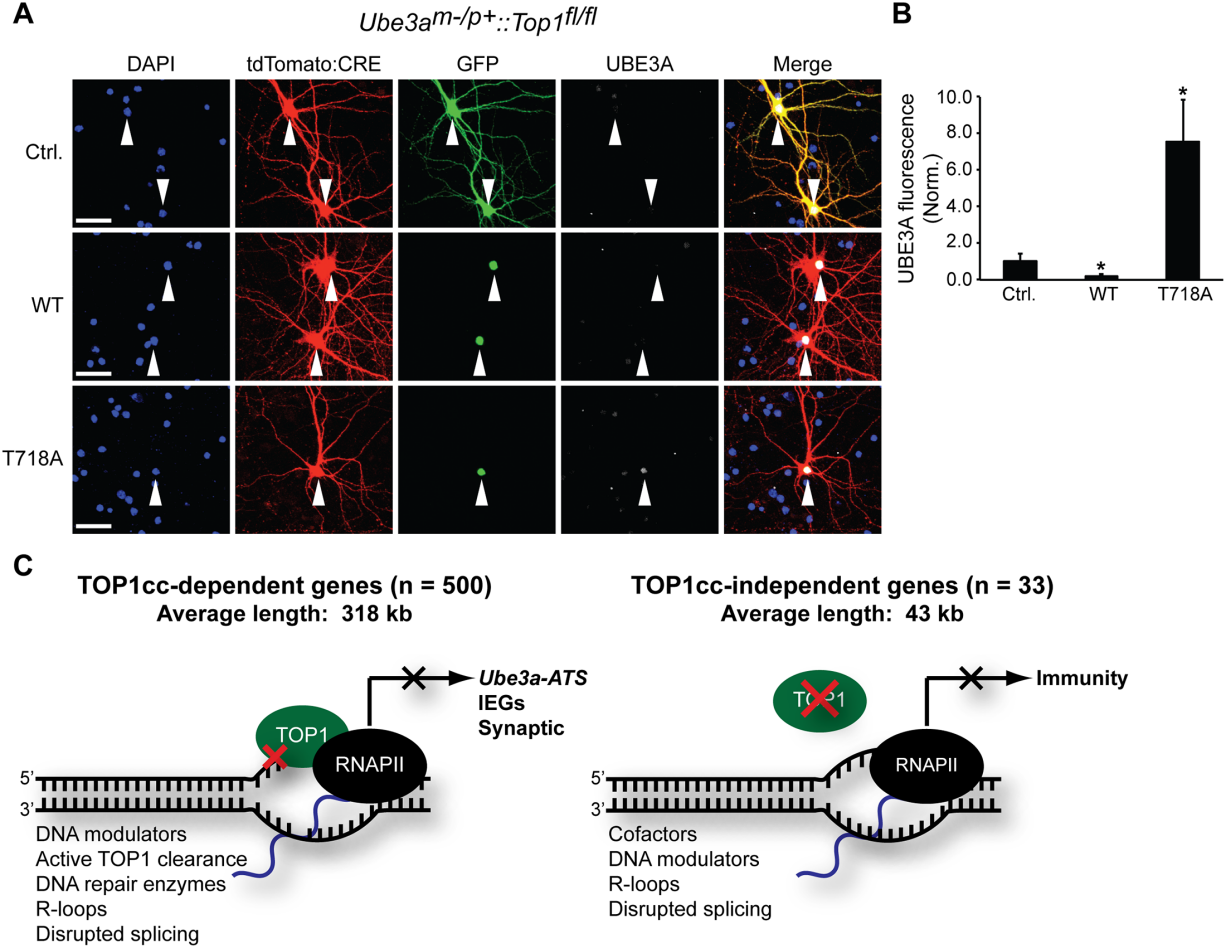
*Ube3a* unsilencing is TOP1cc-dependent. (A) *Ube3a*^*m-/p+*^::*Top1*^*fl/fl*^ (AS) cortical neuron cultures were transfected with tdTomato-P2A-CRE and GFP, GFP-TOP1, or the TOP1 cleavage complex mimetic GFP-TOP1 T718A at DIV 6. Cells were then fixed at DIV 13. (B) Quantification of UBE3A immunostaining. Values are normalized to UBE3A intensity in the GFP control. Mean ± s.e.m., unpaired student’s t-test; * p < 0.05, n = 5 culture sets. (C) Model depicting TOP1 regulation of gene transcription in neurons. Most genes (n = 500) that are downregulated following TOP1 disruption are TOP1cc-dependent and long (on average ~318 kb), while a minority of genes (n = 33) are TOP1cc-independent and short (on average ~43 kb). Expression of some genes (n = 80), are sensitive to TOP1cc-dependent or -independent mechanisms and are exceptionally long (on average ~444 kb). Listed are potential factors that may coordinate with TOP1 to allow for these distinct mechanisms of TOP1-dependent gene regulation.

We further tested the importance of TOP1cc’s in the mechanism of *Ube3a* unsilencing by evaluating how a TOP1 cleavage complex mimetic (T718A) affected *Ube3a* expression. This TOP1 T718A point mutation slows the DNA religation rate of TOP1 and was previously used to address the functional relevance of TOP1cc’s [32,33]. In yeast, this point mutation is lethal [32], but we found that postmitotic cortical neurons tolerated expression for at least 7 days (S6A Fig). In cultured neurons, the TOP1 T718A mutant increased TOP1-DNA covalent complexes compared to GFP or WT TOP1 (S6A and S6B Fig). We co-transfected *AS*::*Top1*^*fl/fl*^ cortical neurons with CRE to selectively delete *Top1* and with plasmids expressing GFP, GFP-TOP1, or GFP-TOP1 T718A (Fig 6A) and then monitored changes in UBE3A protein levels. We found that overexpression of the T718A point mutation upregulated paternal UBE3A, whereas GFP and GFP-TOP1 alone had no effect (Fig 6A and 6B). Taken together, these data suggest that TOP1cc formation can unsilence the paternal copy of *Ube3a*.

## Discussion

Topoisomerases have been extensively studied in the cancer field [2,4], but their contribution to nervous system function is only beginning to emerge. Here, we created a *Top1* cKO mouse to elucidate the mechanisms governing TOP1-dependent gene regulation in postmitotic neurons. Surprisingly, we found that deletion of *Top1* results in down- and upregulation of only a fraction of genes compared to treatment with the TOP1 inhibitor, topotecan (Fig 2C and 2D). Topotecan does not further reduce transcript levels in *Top1* cKO neurons, suggesting that the transcriptional effects of topotecan are dependent on TOP1. We also found that genetic deletion of *Top1* reduced the expression of a subset of long genes, as has been demonstrated previously [22]; however the consequences of genetic deletion were smaller than the effects of topotecan treatment (Fig 2B). Like topotecan, *Top1* deletion decreased protein levels of synaptic adhesion molecules such as NEUREXIN-1, NEUROLIGIN-1, and CNTNAP2 [23], all of which are encoded by extremely long genes. However, unlike in topotecan-treated neurons, we found that *Top1* deletion was not sufficient to unsilence *Ube3a*, nor was it sufficient to decrease the expression of IEGs. Using TOP1 catalytic inhibitors that block TOP1 unwinding activity but do not create TOP1cc’s [28,29], we observed decreased expression of synaptic adhesion proteins but no *Ube3a* unsilencing, even at the highest doses tested. However, expression of a TOP1 cleavage complex mimetic (T718A) was sufficient to unsilence *Ube3a*. Taken together, our findings strongly indicate that TOP1cc’s contribute to repression of the *Ube3a-ATS*, and unsilencing of the paternal *Ube3a* allele in neurons, and may be critical for repression of a multitude of neuronal genes (Fig 6C).

Here we identified two mechanisms underlying TOP1-dependent dysregulation of gene expression in neurons (Fig 6C). 1) Expression of TOP1cc-dependent genes are affected following topotecan treatment but not changed following *Top1* knockout (Fig 6C, left). Most of these differentially expressed genes (n = ~500) require the formation of TOP1cc’s and are long (318 kb on average). An analog of topotecan (camptothecin) likewise forms TOP1cc’s and reduces expression of numerous long genes in mammalian cell lines [24]. 2) Expression of TOP1cc-independent genes are affected in *Top1* cKO neurons but are not affected in WT neurons treated with topotecan (Fig 6C, right). These genes tend to be much smaller in size (~43 kb). Additionally, a third group of genes are sensitive to both of these mechanisms: TOP1 levels or TOP1cc’s. These genes are altered in *Top1* cKO neurons and in topotecan-treated WT neurons, and tend to be exceptionally long (~440 kb, 80 in total) (S4 Table). This gene list contains synaptic adhesion molecules such as *Nlgn1*, *Nrxn1*, and *Cntnap2*.

Using three different genetic approaches (conditional knockout, CRISPR-Cas9 deletion, shRNA knockdown), we found that *Top1* deletion does not significantly increase *Ube3a* expression (Figs 2F, 4C and 5, S3 Fig). In contrast, overexpression of TOP1 T718A, a mutant that stabilizes TOP1cc’s in neurons, did unsilence paternal *Ube3a* (Fig 6A and 6B). And, topotecan, an inhibitor that forms TOP1cc’s, unsilenced paternal *Ube3a*. Inhibitors that do not form TOP1cc’s, including CYB-L10, did not unsilence paternal *Ube3a*. These data strongly suggest TOP1cc formation, and not loss of TOP1, drives paternal *Ube3a* unsilencing. However, other mechanisms besides TOP1cc formation may promote reactivation of paternal *Ube3a*. For example, TOP2 inhibitors unsilence paternal UBE3A [13], although whether these inhibitors stabilize TOP1cc’s in neurons is unknown.

Additional mechanisms are known to participate in TOP1-dependent gene regulation. For example, TOP1 promotes efficient transcription by resolving DNA supercoiling, which minimizes R-loop (DNA:RNA hybrids) formation [4]. Deletion of *Top1* leads to R-loop formation and impairment of gene transcription [34]. TOP1 inhibitors that form cleavable complexes increase R-loops in neurons [35,36], and R-loop formation is implicated in unsilencing the paternal *Ube3a* allele [35]. One could envisage a model where excessive R-loops created by stalled TOP1cc’s shut down transcription in neurons. Whether more R-loops are formed following TOP1cc formation relative to *Top1* deletion is unknown. Although, given that *Top1* deletion did not unsilence *Ube3a*, our data suggest that any R-loops that are formed following *Top1* deletion may not be sufficient to fully block long gene transcription and *Ube3a-ATS*. TOP1cc’s may also be required to facilitate this downregulation.

Topoisomerase cleavage complexes can be converted into DNA double strand breaks and in some cases, serve as a mechanism to initiate transcription [37,38]. In neurons, inhibition of *Top2β* with etoposide increases the expression of IEGs by generating DNA double strand breaks and recruiting transcriptional coactivators [39]. In our present study, and in previous work [40], we found that topotecan decreased IEG expression in neuronal cultures. This is the opposite of what was observed following *Top2β* inhibition. Moreover, we found that deletion of *Top1* is not sufficient to decrease IEG expression, suggesting that the formation of TOP1cc’s downregulate IEG expression. Alternatively, decreased IEG expression might reflect an indirect consequence of reduced spontaneous neuronal activity, which occurs following topotecan treatment [23].

Intriguingly, the transcriptome of neurons is biased for longer genes relative to non-neuronal cell types [41–43], and this length bias is more pronounced in some brain regions like prefrontal cortex and amygdala over other regions [41]. Moreover, these long genes are involved in neurotransmission and synaptic function—processes that are uniquely important to neurons. Our findings raise the possibility that neurons might be particularly vulnerable to transcriptional deficits that originate from TOP1cc’s or TOP1 deletion.

Stalled TOP1cc’s can recruit factors that physically remove TOP1 from DNA [37,44–46]. These factors include ATM, a master DNA repair protein, and DNA-PK, which both regulate ubiquitin-dependent turnover of TOP1 [45,47]. In the absence of these two factors, TOP1cc’s accumulate in neurons. Misregulation of TOP1 has been observed in neurodegenerative disorders [45,48,49] and missense mutations and disruptions of genes that regulate TOP1 have been identified in individuals with autism spectrum disorders [22,50–52]. Thus, changes in TOP1cc’s and TOP1 levels could contribute to a multitude of neurological disorders.

## Supporting Information

S1 Fig Validation of *Top1* cKO. Cortical neurons were infected with tdTomato or tdTomato-P2A-CRE lentivirus at DIV 3 and then were harvested at DIV 7 and DIV 10. Representative immunoblots for rabbit anti-TOP1 and mouse anti-TOP1. ACTIN was used as a loading control. Molecular weight markers are shown on the right.
(TIF)

S2 Fig Topotecan reduces expression of immediate early genes (IEGs) in a *Top1*-dependent manner but deletion of *Top1* alone is not sufficient to reduce expression of IEGs. (A—H) Quantification of transcript level changes from RNA-seq. Normalized RPKM values relative to WT-Veh. Mean ± s.e.m. FDR < 0.1, n = 3 cultures.
(TIF)

S3 Fig TOP1 depletion by shRNA reduces synaptic adhesion protein expression but does not unsilence *Ube3a*. (A) Cortical neuron cultures were infected with scrambled (Scr) control or *Top1*-shRNA lentiviruses at DIV 3. Neurons were harvested at DIV 10. Representative immunoblots for NRXN1, NLGN1, UBE3A, TOP1, and ACTIN. (B) Quantification of fold change in protein expression normalized to ACTIN. Mean ± s.e.m., unpaired student’s t-test; * p < 0.05, n = 4 cultures. (C) *Ube3a*^*m-/p+*^ (AS) cortical neuron cultures were infected with either Scr control or *Top1*-shRNA at DIV 3. Neurons were harvested at DIV 10. Representative immunoblots for UBE3A, TOP1, and ACTIN. (D) Quantification of fold change in protein expression normalized to ACTIN. Mean ± s.e.m., unpaired student’s t-test; * p < 0.05, n = 3 cultures.
(TIF)

S4 Fig Top1 deletion is not sufficient to unsilence *Ube3a*. (A) Zoomed in images of WT (top) and AS (bottom) cortical neuron cultures were transfected with tdTomato and Cas9 alone (Ctrl.) or Cas9 and a sgRNA directed to *Top1*. Scale bar, 10 μm.
(TIF)

S5 Fig TOP1 catalytic inhibitors do not unsilence *Ube3a* but do reduce expression of synaptic adhesion proteins. (A) Structures of TOP1 catalytic inhibitors used to test *Ube3a* unsilencing. (B) *Ube3a*^*wt/YFP*^ cortical neuron cultures were treated with Vehicle (Veh), the catalytic TOP1 inhibitor CYB-L10, or topotecan at DIV 7 for 72 hours. Scale bar, 100 μm. (C) Dose response curve for UBE3A-YFP paternal unsilencing following treatment with topotecan, CY08C, CY13B, CYB-L01, or CYB-L10. (D) Cortical neuron cultures were treated with Vehicle (Veh), the catalytic TOP1 inhibitor CYB-L10, or topotecan at DIV 7 for 72 hours. Representative immunoblots for NRXN1, NLGN1, CNTNAP2, UBE3A, and ACTIN. (E) Quantification of fold change in protein expression normalized to ACTIN. Mean ± s.e.m., unpaired student’s t-test; * p < 0.05, n = 3.
(TIF)

S6 Fig TOP1 T718A point mutation increases TOP1cc’s in primary cortical neurons. (A) WT cortical neuron cultures were transfected with tdTomato and GFP, GFP-TOP1, or the TOP1 cleavage complex mimetic GFP-TOP1 T718A at DIV 6. Cells were then fixed at DIV 13. TOP1cc intensity is shown using the Fire Lookup Table in FIJI. Scale bar, 50 μm. Zoomed inset scale bar, 10 μm. (B) Quantification of TOP1cc immunostaining. Mean ± s.e.m., unpaired student’s t-test; * p < 0.05, n = 9 cells per condition.
(TIF)

S1 Table Fold change in transcript levels in WT-Topot, *Top1* cKO-Veh, and *Top1* cKO-Topot relative to WT-Veh cortical neuron cultures. Shown is a list of significant downregulated and upregulated genes in each condition along with raw RPKM values from each condition.
(XLSX)

S2 Table Functional annotation of significant downregulated genes in each condition using DAVID analysis. (XLSX)

S3 Table Functional annotation of significant upregulated genes in each condition using DAVID analysis. (XLSX)

S4 Table List of Top1cc-dependent, Top1cc-independent, and Top1cc-dependent or -independent TOP1 downregulated genes and their functional annotations. Graph of average gene length of downregulated genes in the three classes listed above.
(XLSX)
