# Supplementary figures and images for: Topoisomerase 1 Regulates Gene Expression in Neurons through Cleavage Complex-Dependent and -Independent Mechanisms
Source: PLoS One. 2016 May 27;11(5):e0156439. doi: 10.1371/journal.pone.0156439 (PMC4883752; doi:10.1371/journal.pone.0156439)

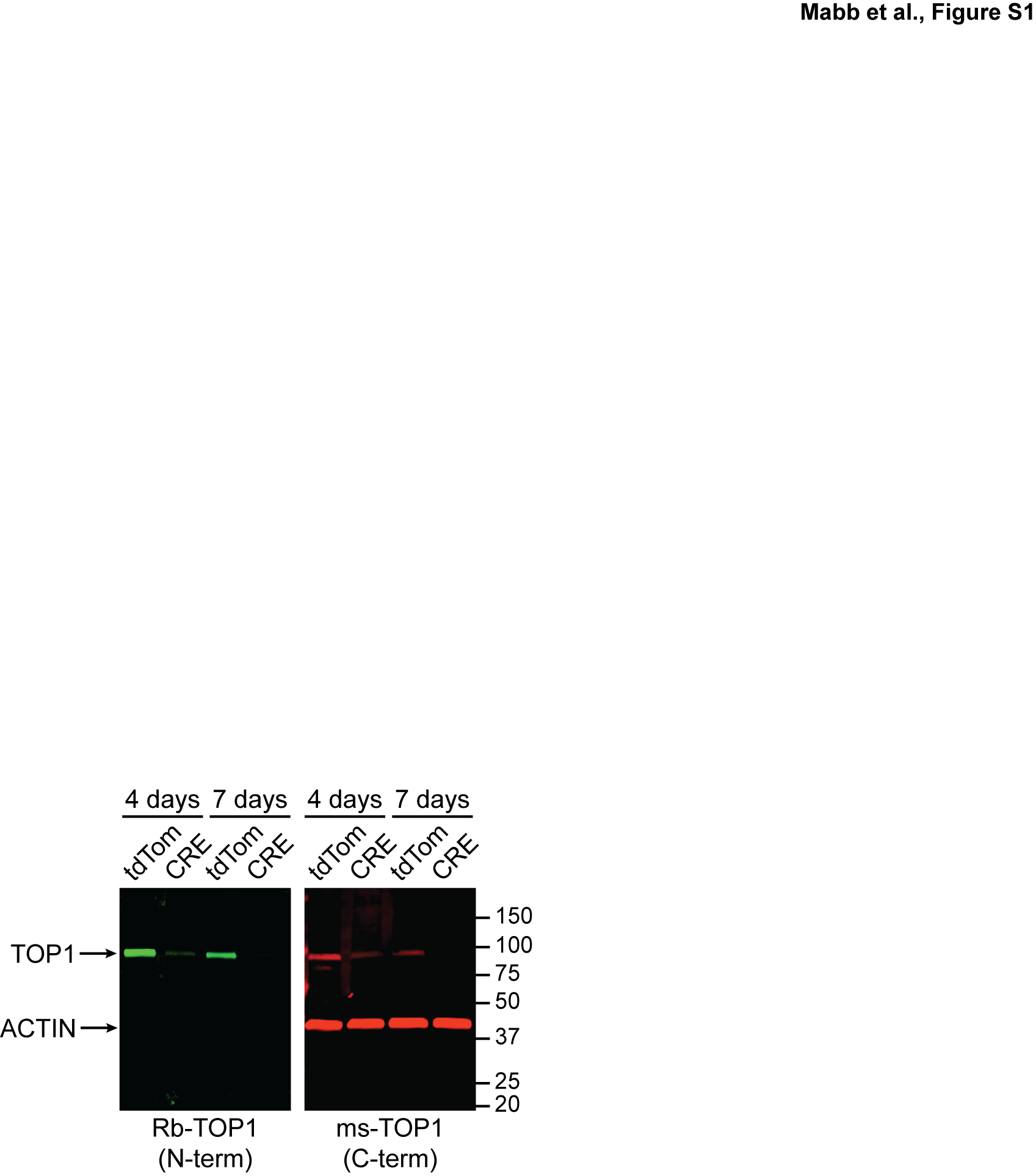

Supplement: S1 Fig — Cortical neurons were infected with tdTomato or tdTomato-P2A-CRE lentivirus at DIV 3 and then were harvested at DIV 7 and DIV 10. Representative immunoblots for rabbit anti-TOP1 and mouse anti-TOP1. ACTIN was used as a loading control. Molecular weight markers are shown on the right. (TIF) [file pone.0156439.s001.tif]

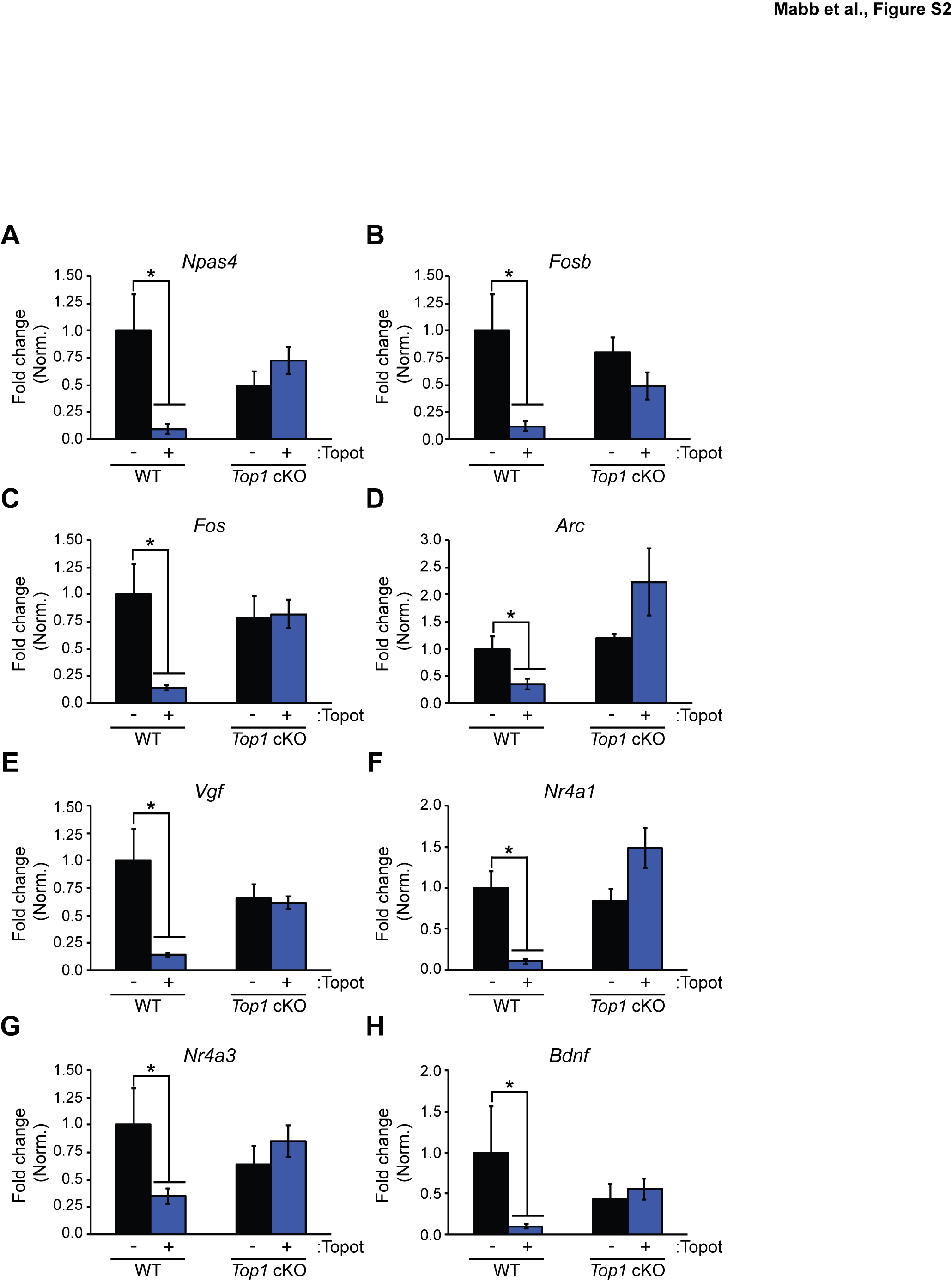

Supplement: S2 Fig — (A—H) Quantification of transcript level changes from RNA-seq. Normalized RPKM values relative to WT-Veh. Mean ± s.e.m. FDR < 0.1, n = 3 cultures. (TIF) [file pone.0156439.s002.tif]

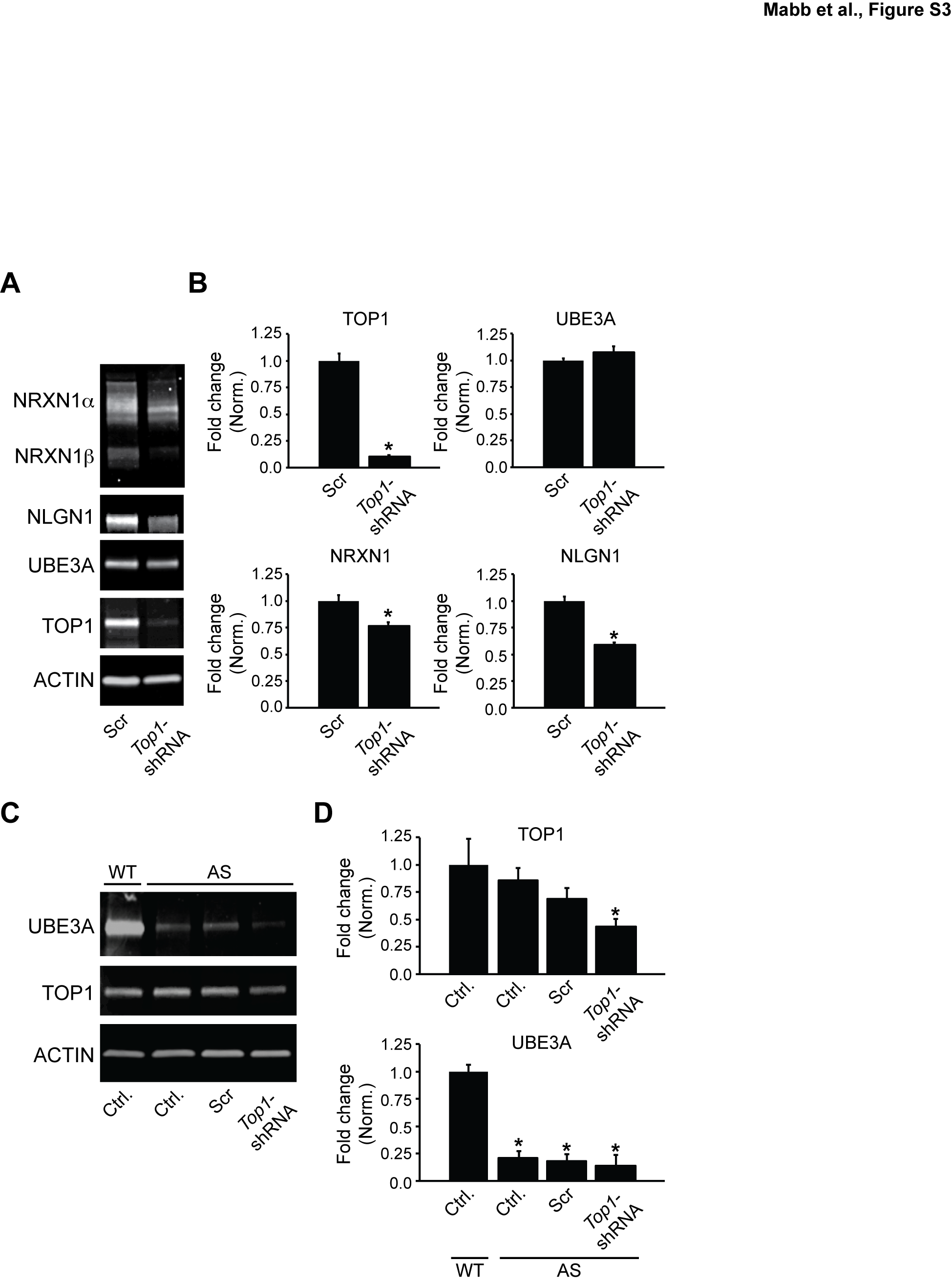

Supplement: S3 Fig — (A) Cortical neuron cultures were infected with scrambled (Scr) control or Top1-shRNA lentiviruses at DIV 3. Neurons were harvested at DIV 10. Representative immunoblots for NRXN1, NLGN1, UBE3A, TOP1, and ACTIN. (B) Quantification of fold change in protein expression normalized to ACTIN. Mean ± s.e.m., unpaired student’s t-test; * p < 0.05, n = 4 cultures. (C) Ube3am-/p+ (AS) cortical neuron cultures were infected with either Scr control or Top1-shRNA at DIV 3. Neurons were harvested at DIV 10. Representative immunoblots for UBE3A, TOP1, and ACTIN. (D) Quantification of fold change in protein expression normalized to ACTIN. Mean ± s.e.m., unpaired student’s t-test; * p < 0.05, n = 3 cultures. (TIF) [file pone.0156439.s003.tif]

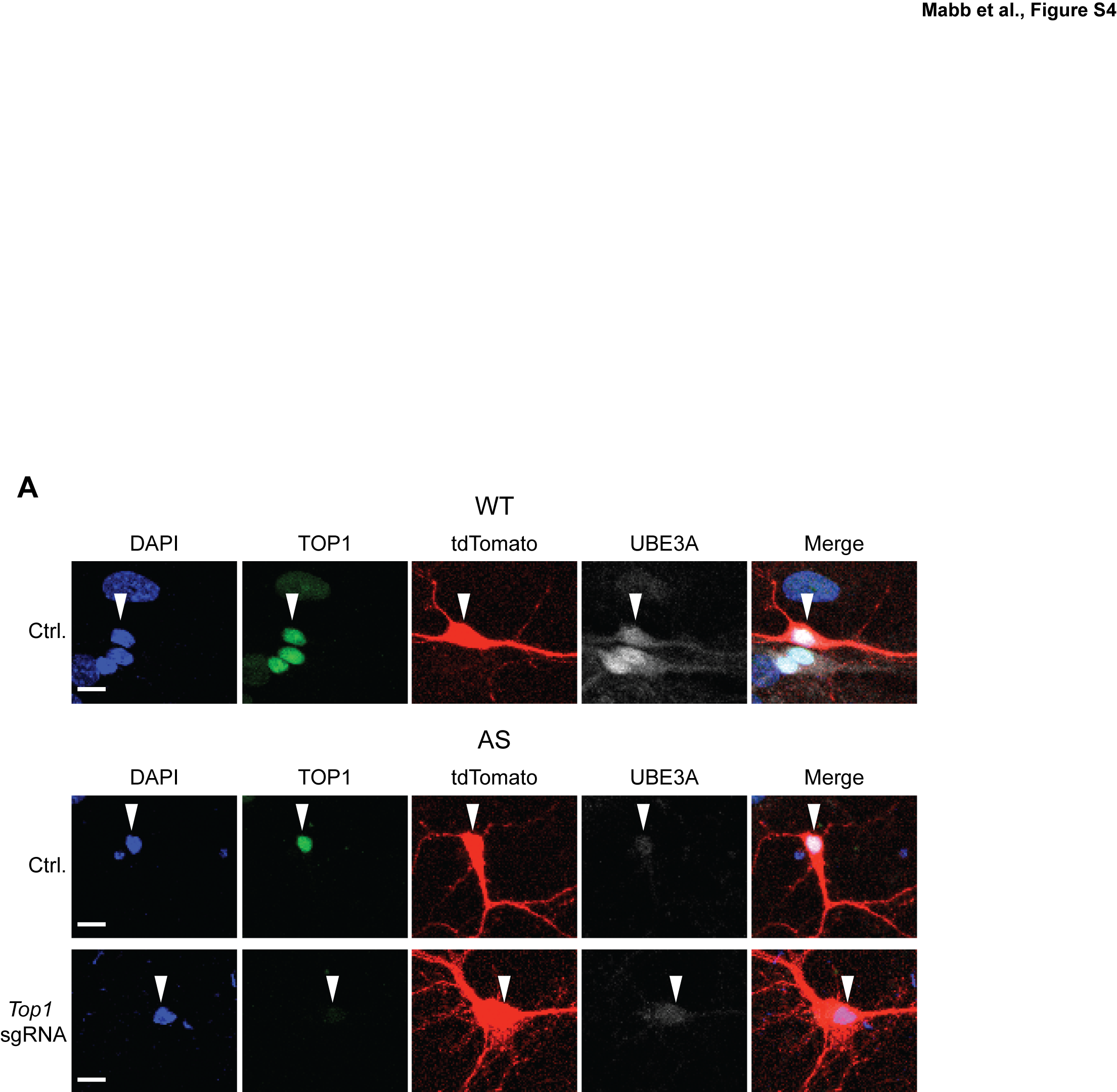

Supplement: S4 Fig — (A) Zoomed in images of WT (top) and AS (bottom) cortical neuron cultures were transfected with tdTomato and Cas9 alone (Ctrl.) or Cas9 and a sgRNA directed to Top1. Scale bar, 10 μm. (TIF) [file pone.0156439.s004.tif]

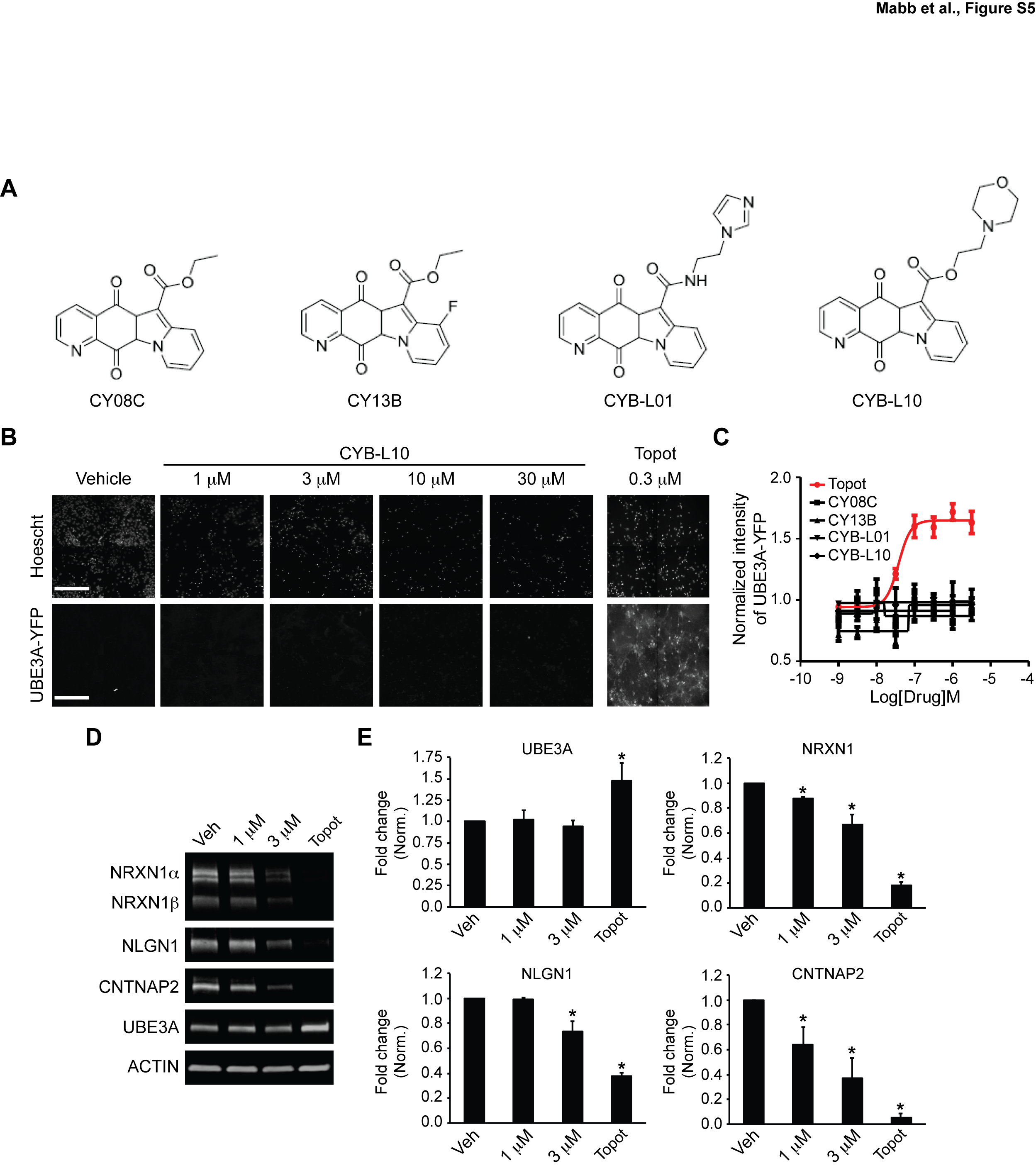

Supplement: S5 Fig — (A) Structures of TOP1 catalytic inhibitors used to test Ube3a unsilencing. (B) Ube3awt/YFP cortical neuron cultures were treated with Vehicle (Veh), the catalytic TOP1 inhibitor CYB-L10, or topotecan at DIV 7 for 72 hours. Scale bar, 100 μm. (C) Dose response curve for UBE3A-YFP paternal unsilencing following treatment with topotecan, CY08C, CY13B, CYB-L01, or CYB-L10. (D) Cortical neuron cultures were treated with Vehicle (Veh), the catalytic TOP1 inhibitor CYB-L10, or topotecan at DIV 7 for 72 hours. Representative immunoblots for NRXN1, NLGN1, CNTNAP2, UBE3A, and ACTIN. (E) Quantification of fold change in protein expression normalized to ACTIN. Mean ± s.e.m., unpaired student’s t-test; * p < 0.05, n = 3. (TIF) [file pone.0156439.s005.tif]

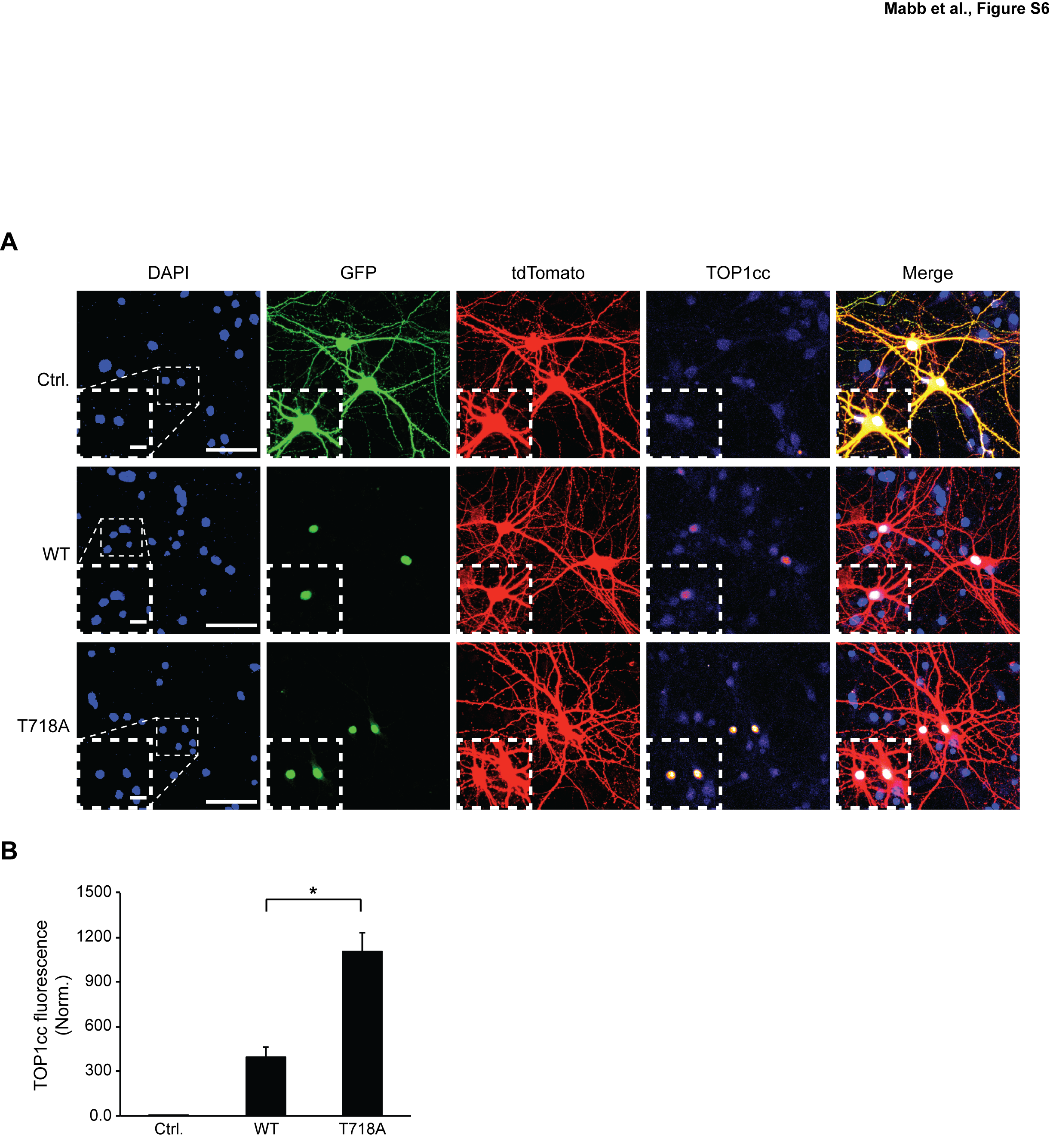

Supplement: S6 Fig — (A) WT cortical neuron cultures were transfected with tdTomato and GFP, GFP-TOP1, or the TOP1 cleavage complex mimetic GFP-TOP1 T718A at DIV 6. Cells were then fixed at DIV 13. TOP1cc intensity is shown using the Fire Lookup Table in FIJI. Scale bar, 50 μm. Zoomed inset scale bar, 10 μm. (B) Quantification of TOP1cc immunostaining. Mean ± s.e.m., unpaired student’s t-test; * p < 0.05, n = 9 cells per condition. (TIF) [file pone.0156439.s006.tif]
